# Heparanase promotes neuroinflammatory response during subarachnoid hemorrhage in rats

**DOI:** 10.1186/s12974-017-0912-8

**Published:** 2017-07-18

**Authors:** Benjarat Changyaleket, Zhao Zhong Chong, Randal O. Dull, Danop Nanegrungsunk, Haoliang Xu

**Affiliations:** 1 Department of Anesthesiology, University of Illinois at Chicago, Chicago, IL USA; 2 Department of Pathology, University of Illinois at Chicago, 840 South Wood Street, Chicago, IL 60612 USA

**Keywords:** Heparanase, Inflammation, Subarachnoid hemorrhage, Neurological function

## Abstract

**Background:**

Heparanase, a mammalian endo-β-D-glucoronidase that specifically degrades heparan sulfate, has been implicated in inflammation and ischemic stroke. However, the role of heparanase in neuroinflammatory response in subarachnoid hemorrhage (SAH) has not yet been investigated. This study was designed to examine the association between heparanase expression and neuroinflammation during subarachnoid hemorrhage.

**Methods:**

Rats were subjected to SAH by endovascular perforation, and the expression of heparanase was determined by Western blot analysis and immunofluorescence in the ipsilateral brain cortex at 24 h post-SAH. Pial venule leukocyte trafficking was monitored by using intravital microscopy through cranial window.

**Results:**

Our results indicated that, compared to their sham-surgical controls, the rats subjected to SAH showed marked elevation of heparanase expression in the ipsilateral brain cortex. The SAH-induced elevation of heparanase was accompanied by increased leukocyte trafficking in pial venules and significant neurological deficiency. Intracerebroventricular application of a selective heparanase inhibitor, OGT2115, which was initiated at 3 h after SAH, significantly suppressed the leukocyte trafficking and improved the neurological function.

**Conclusions:**

Our findings indicate that heparanase plays an important role in mediating the neuroinflammatory response after SAH and contributes to SAH-related neurological deficits and early brain injury following SAH.

## Background

Subarachnoid hemorrhage (SAH) is a devastating disease. Although it accounts for only a small percentage (5%) of overall stroke, SAH is associated with a high mortality rate that reaches up to 50% [1]. In addition, those who survive the initial bleeding may experience permanent, severe neurological deficits, excluding them from the workforce [1]. Since approximate 50% of SAH occurs in patients younger than 55 years of age, the disease poses a great economic burden in society. As a result, identifying therapeutic strategies to treat SAH and its complications is of extreme importance.

Neuroinflammation is believed to play a key role in the poor outcome of SAH [2], but its exact mechanisms are not well defined. Delayed cerebral ischemia (DCI) is a major complication of SAH characterized by inflammation, cerebral edema, and microthrombosis, collectively leading to brain infarction and neurological deficits, typically appearing 3 days after SAH [3]. Cerebral vasospasm was considered to be a major mechanism of DCI [2, 4] with inflammation as a key factor contributing to the induction of vasospasm following SAH [5]. For example, proinflammatory cytokine levels in the brain 4–6 days following SAH correlates with the severity of radiographically proven vasospasm [6]. By contrast, inhibitors of these cytokines attenuate the SAH-induced vasospasm [7–11]. In addition, general anti-inflammatory treatment using corticosteroids has been shown to reduce vasospasm, attenuate delayed cerebral ischemia, and improve outcomes in both experimental and clinical SAH [12–15].

Heparanase is emerging as a potential candidate that regulates neuroinflammation. Heparanase is a heparan sulfate degrading enzyme that cleaves heparan sulfate (HS) on the HS proteoglycans (HSPG) and has been implicated in tumor metastasis and inflammation [16]. Heparanase is extensively expressed in many cells, including neurons, astrocytes, endothelial cells, and macrophages [17–21]. Heparanase is synthesized and secreted as a latent 65 kDa precursor and is retained at pericellular sites binding to cell surface HS components [22, 23]. Acidic environments as observed in tumor or inflammatory conditions are optimal for the enzymatic activity [24–26]. In contrast, heparanase expressed on the surface of cells do not enzymatically degrade HS in tissues with normal physiologic conditions [27]. HSPG is a major component of the extracellular matrix (ECM). Thus, the cleavage of HSPG may regulate the remodeling of the ECM and allow for tissue invasion of circulating cells and/or leukocyte trafficking across the endothelium, a critical process in inflammation. Inhibition of heparanase was shown to be effective in reducing inflammation [28–31].

The information concerning the role of heparanase in neuroinflammation during different types of strokes is limited. A few reports have demonstrated that heparanase is upregulated in the rat brain after ischemic stroke [16, 19, 32]. No data is available regarding the association between heparanase and SAH. Our current study examined heparanase expression in the rat cerebral cortex in the early phase of brain injury post SAH. We also examined the effect of inhibiting heparanase function on neurological outcome and leukocyte trafficking in rats. Our findings illustrate that heparanase expression is increased after SAH and inhibition of heparanase attenuates SAH-induced neurovascular inflammation and improves neurological function after SAH.

## Methods

### Animal preparation and procedures

The protocol was approved by the University of Illinois Institutional Animal Care and Use Committee. Male Sprague-Dawley rats, weighing 250–300 g (Charles River, Wilmington, MA), were randomly assigned to undergo sham surgery (group 1), SAH surgery (group 2), SAH surgery + vehicle (group 3), or SAH surgery + heparanase inhibitor (group 4). In groups 1 and 2, we examined the effect of SAH on heparanase expression and determined its localization to specific cell types in the brain via western blot analysis (*n* = 4) and immunofluorescence stain (*n* = 6). These animal groups were sacrificed at 24 h post sham/SAH surgeries. In groups 3 and 4, we tested the hypothesis that inhibiting endogenous heparanase activity, using heparanase inhibitor, OGT2115, prevents post-SAH neuroinflammation (*n* = 4–6) and improves neurologic outcome (*n* = 6). Three hours after SAH induction, animals in these two groups received either a vehicle treatment or OGT2115 via the osmotic pump. Neurological scores were assessed at 48 h post-SAH, followed by pial venule intravascular leukocyte adhesion (PVLA) evaluation via a cranial window-intravital microscopy system. An additional sham surgical group that is identical to group 1 (*n* = 6) was added to this study for better comparison.

### Subarachnoid hemorrhage (SAH) model

The details of SAH endovascular perforation model were previously described [33]. Briefly, rats weighing 250–300 g were anesthetized with 70% N_2_O/30% O_2_/2% isoflurane using a rodent ventilator. The tail artery was cannulated to monitor arterial pressure. Blood pressure, blood gases, and body temperature were recorded and kept within normal ranges by adjusting ventilating rate and using a servo-controlled heating pad. Regional cerebral blood flow (rCBF) over the middle cerebral artery (MCA) territory was continuously monitored before, during, and 30 min after SAH via laser-Doppler flowmetry (LDF).

To induce SAH, the right external carotid artery (ECA), common carotid artery (CCA), and internal carotid artery (ICA) were exposed. After the CCA were temporarily occluded, a polytetrafluoroethylene tube was inserted into the ICA via the ECA until resistance was felt. SAH was induced by a 2-mm protrusion of a tungsten wire inside tube, puncturing the proximal anterior cerebral artery. After retracting the wire and tubing, the CCA was reopened to allow reperfusion via the ICA. The arterial catheter was removed, the wounds were closed, and anesthesia was discontinued. When spontaneous breathing was reestablished, the rats were extubated and returned to their cages. This modified SAH model has shown high reproducibility. However, maintaining blood gas and blood pressure among all experimental groups, as well as puncturing the appropriate intracranial vascular site, is important to minimize the variation of intracranial hemorrhage. At the end of the experiment, the presence of SAH was confirmed by direct observation of blood accumulation around the proximal anterior cerebral artery where the perforation occurred. Based on our previous findings, successful SAH showed remarkable blood accumulation within the first 24 h that would then decrease in size. In most cases, blood accumulation could no longer be detected after 72 h post-SAH (data not shown).

### Alzet osmotic pump installation

Three hours after SAH/sham surgeries, the Alzet osmotic pumps (Model 1003D, Alzet, Cupertino, CA) were installed subcutaneously in the neck region to provide the animals either vehicle or heparanase inhibitor, OGT2115 (MW: 495.3, Tocris Bioscience, Cat. #2710). The method has been described previously in details [34]. Briefly, rats were placed in a stereotactic frame under isoflurane anesthesia. The skull was exposed and a small hole over the right lateral ventricle (8 mm caudal to bregma, 1.5 mm lateral to midline, and 4 mm beneath the skull surface) was made to allow intracerebroventricular drug delivery. An initial loading dose of OGT2115 [0.05 μg in 5 μl artificial cerebrospinal fluid (aCSF)] or 5 μl vehicle (aCSF) was injected slowly into the lateral ventricle, followed by implantation of an infusion cannula with attached osmotic pump. This provided a continuous drug delivery at a rate of 1 μL/h to maintain CSF concentration of OGT2115 at approximate 0.4 μM, which is its IC50 for heparanase inhibition [35]. The OGT2115 was administered continuously until animals were sacrificed at 48 h.

### Cranial window and intravital microscopy

To assess leukocyte trafficking, the cranial windows were installed at 48 h after SAH as previously described [36]. Briefly, the animals were intubated and mechanically supported under 70% N_2_O/30%O_2_/2% isoflurane. Femoral artery and vein were cannulated for monitoring arterial blood pressure and for obtaining samples for arterial blood gases. Body temperature was kept at 37 °C. A 10-mm craniotomy was performed midline over the skull. After removing the dura, a 11-mm diameter glass window equipped with three ports (inflow, outflow, and intracranial pressure monitoring) was glued to the skull with cyanoacrylate. The intracranial pressure (ICP) under the cranial window was monitored and maintained within 7 to 11 mmHg range, by adjusting the height of the outflow port, throughout the experiment. After completion of the surgery, the anesthesia with isoflurane was switched to fentanyl infusion for monitoring of rhodamine-6G (R6G)-labeled leukocyte. This semiquantitative method of in vivo assessment of leukocyte trafficking has been described in details in our previous publications [33, 37]. Briefly, loading dose of R6G (0.2 mg) was applied intravenously, followed by 0.2 mg/h maintenance dose. Pial venules (approximate 40 to 60 μm in diameter) were randomly selected for study. Images of R6G-labeled leukocytes were captured using a digital camera (CoolSnap ES, Photometrics, Tucson, AZ, USA) mounted on a fluorescence microscope (Nikon, Melville, NY, USA) and analyzed using MetaMorph software (Molecular Devices, Downingtown, PA, USA). Leukocyte adhesion was reported as the percentage of adherent leukocytes (this includes both tightly adhered and slow-rolling leukocytes) occupying the viewed venular area in the microscopic field and was used as a surrogate marker of neuroinflammation [37].

### Western blots

Brain cortical homogenate was centrifuged and the supernatant collected for protein determination (Bradford method). Western blot analysis of protein expression changes of heparanase was performed according to a protocol previously described [38]. Briefly, the blots were hybridized with rabbit anti-heparanase polyclonal antibody (1:1000, MyBioSource, Inc., San Diego, CA) and subsequently incubated with donkey anti-rabbit IgG secondary antibody conjugated with infrared fluorescent dye (IRDye 800 from LI-COR technology, Lincoln, NE) diluted 1:10,000 in 0.5× Odyssey blocking buffer and 0.1% Tween 20. β-actin was used as a loading control (anti β-actin rabbit monoclonal, 1:1000, Cell Signaling Technology Inc., Boston, MA). Blot membranes were then scanned using the LI-COR Odyssey Infrared Imaging System (LI-COR Biosciences, Lincoln, NE). The protein level of heparanase in each brain sample (*n* = 4 in each group) was expressed as the ratio of the optical densities of heparanase and β-actin bands.

### Immunohistochemistry

Twenty-four hours after surgery, rats were sacrificed and transcardially perfused with cold phosphate-buffered saline (PBS) followed by 20 ml paraformaldehyde (4%). Brains were harvested and paraffin-embedded. Coronal sections (8 μm) were obtained and prepared for immunofluorescence staining. The primary antibodies were used at the following dilution: 1:200 for rabbit polyclonal anti-heparanase (MyBiosource, San Diego, CA) and 1:100 for mouse monoclonal anti-NeuN (EMD Millipore, CA). After overnight incubation, sections were washed and incubated for 1 h at 37 °C with the secondary antibodies which were donkey anti-rabbit rhodamine red-X and donkey anti-mouse conjugated with FITC. Nuclei were stained with 400 ng/ml DAPI. Image acquisition was obtained on a Zeiss Axioplan2 fluorescence microscope quipped with an Axiocam MRm digital camera and Axiovision 4.5 imaging software. Images were taken from sections of ipsilateral parietal cortex stained simultaneously and exposed to identical conditions and amount of time, allowing the comparison of fluorescence intensity among regions of interest. Acquisition settings were calibrated for the most optimal signal to noise ratio, and subsequent images were taken under identical settings.

### Neurological assessments

Neurological assessments were performed in animals at 48 h post-SAH by a blinded observer. The assessments were based on a 21-point scale as previously described [33]. The ratings were taken from a sum of scores from seven categories: (i) spontaneous activity, (normal = 3, akinesia = 0); (ii) side stroking (bilateral brisk response = 3, no response = 0); (iii) vibrissae touch (bilateral brisk response = 3, no response = 0); (iv) limb symmetry (forelimb and hindlimb extended = 3, contralateral forelimb and hindlimb completely flexed = 0); (v) lateral turning (bilateral turning ≥ 45° = 3, no turning = 0); (vi) forelimb walking (briskly walks forward in symmetry = 3, cannot move on forelimbs = 0); and (vii) slope climbing (climbs to the top of slope with prompting and strong grip = 3, weak grips and falls off = 0).

### Statistics

Results were presented as means ± SEM. A *p* < 0.05 was considered as statistically significant. Several statistical analyses were used. Protein expression and leukocyte adhesion were analyzed using one-way analysis of variance (ANOVA) combined with a post hoc Tukey’s analysis. Non-parametric neurological behavioral data were analyzed using Mann–Whitney rank sum test. The result was expressed as medians and ranges (boxplot design).

## Results

### Physiological variables

Physiologic parameters including mean arterial pressure (MAP), temperature, and arterial blood gases were maintained within normal physiologic range. In the SAH animals, there was a transient drop in MAP of ~30 mmHg at the initiation of MCA perforation which resolved within 5 min. rCBF also decreased abruptly to 10–20% of the initial baseline at the time of perforation, followed by a gradual recovery to 60% of baseline values within 20 min (Table 1). SAH was also confirmed by post-mortem inspection of the presence of blood accumulation around the proximal anterior cerebral artery where the perforation occurred [37]. No significant differences were observed when comparing temperature, PaCO_2_, pH, and MABP values at equivalent experimental time points among all experimental groups. The mortality rate was 33% in the SAH groups. All animals died within the first 24 h after SAH initiation. No animals died in the sham group.

**Table 1:** Subarachnoid hemorrhage (SAH)-induced cerebral blood flow changes in rats

Subgroup	Number of animals	CBF changes (% of the initial values)	
		1 min post-SAH	20 min post-SAH
Sham surgery (group 1)	16	90.3 ± 7.7	103.2 ± 6.1
SAH surgery (group 2)	10	14.6 ± 3.5*	64.2 ± 5.9*
SAH surgery + vehicle (group 3)	6	16.9 ± 5.2*	62.3 ± 9.0*
SAH surgery + heparanase inhibitor (group 4)	6	15.3 ± 5.7*	58.2 ± 4.6*

Cerebral blood flow (CBF) changes were measured throughout SAH surgical procedure. CBF changes were calculated for 30 min post-SAH and expressed as percentage of initial CBF values. SAH was accompanied with an abrupt CBF drop, followed by a gradual recovery toward its initial value and reached a plateau after 20 min. Three hours post-SAH, animals in groups 3 and 4 received intracerebroventricular treatment of either vehicle (aCSF) or heparanase inhibitor (OGT2115) via Alzet osmotic pump installation. Values are mean ± SE

*p < 0.05 vs the sham surgical controls

### The expression of brain heparanase after SAH

**Fig. 1:**
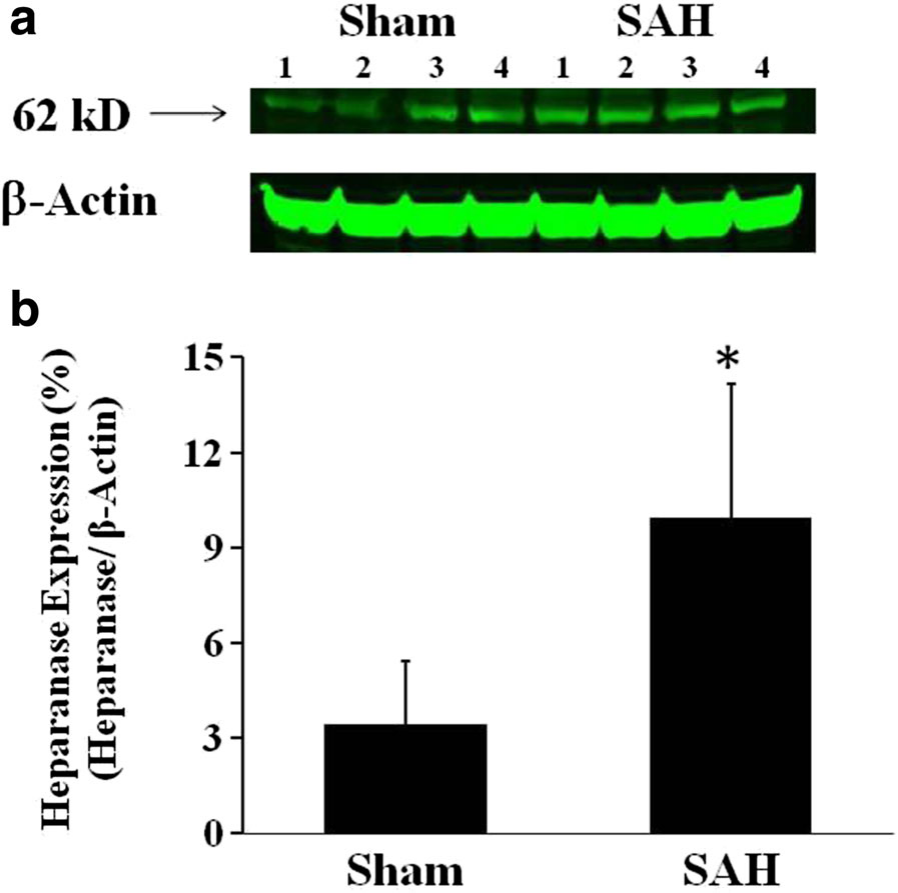
The expression of heparanase increases in cerebral cortex after subarachnoid hemorrhage (SAH) in rats. Western blot was performed for heparanase in the ipsilateral cerebral cortex at 24 h following SAH. **a** Representative images showing the expression of heparanase to be increased at 24 h after SAH. **b** The quantitative results demonstrated that the expression ratio of heparanase/β-actin in density was significantly increased at 24 h after SAH. In **b**, values are mean ± SEM, *n* = 4, *p < 0.05 *vs* sham

**Fig. 2:**
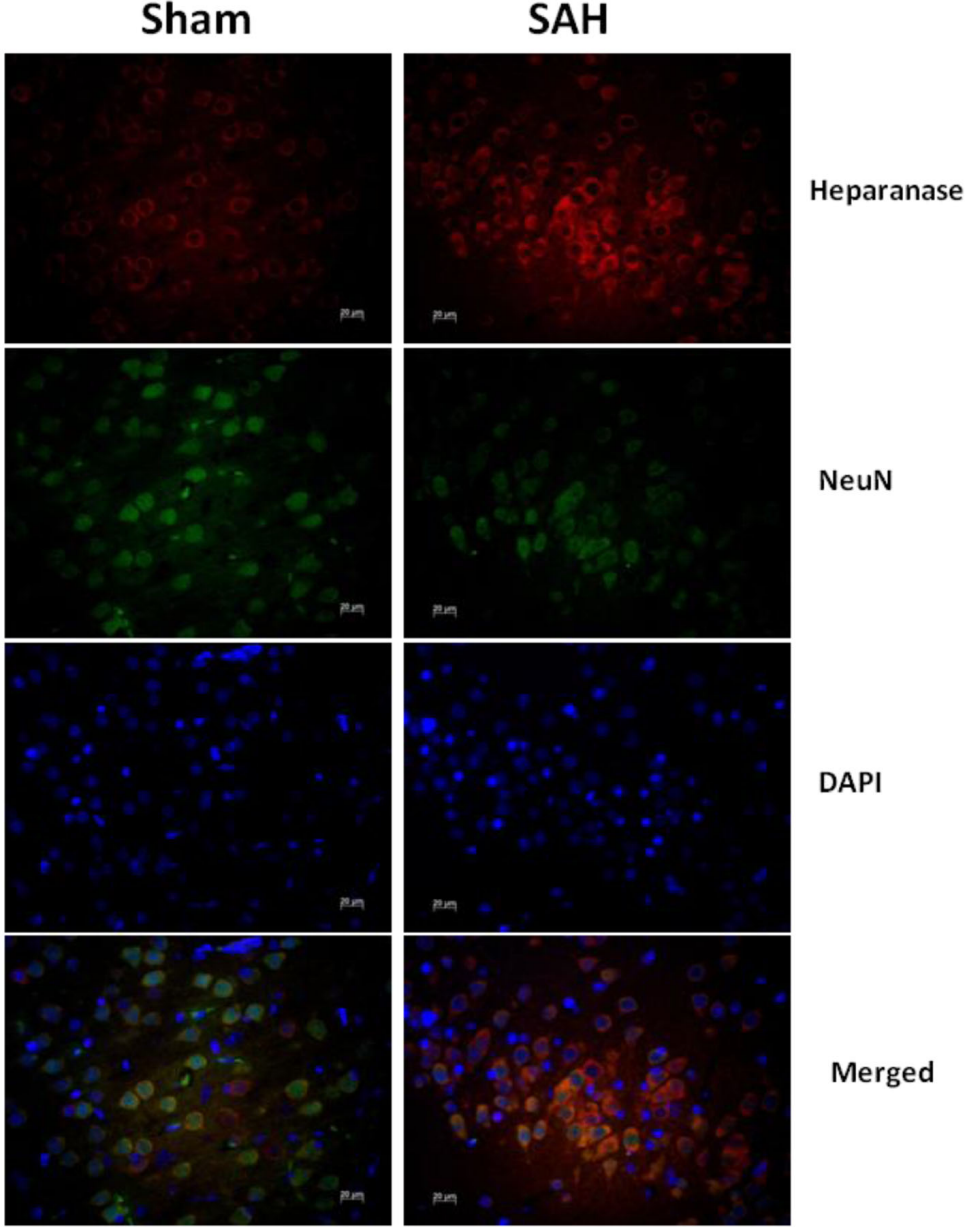
Representative pictures show upregulation of heparanase expression induced by subarachnoid hemorrhage (SAH) in rats from the SAH group compared to their sham controls. Tri-color staining for heparanase (*red*), DAPI (*blue*), and NeuN (*green*) were performed in coronal sections of rat ipsilateral cortex at 24 h post-SAH

To investigate the expression of heparanase in the brain after SAH, Western blot was performed for heparanase in the protein extract of ipsilateral cortex in rats at 24 h following SAH (Fig. 1). SAH was accompanied by a significant elevation of heparanase expression, compared to their matched sham controls (SAH: 9.96 ± 4.1 vs Sham: 3.42 ± 2.0, *p* < 0.05; values were expressed as the ratio of the optical densities of heparanase and β-actin). The result was confirmed by immunofluorescence stain for heparanase, which showed a substantial increase of heparanase expression in the SAH-ipsilateral brain cortex when compared to the sham surgical controls (representative pictures are shown in Fig. 2).

### Heparanase inhibition attenuates pial venular leukocyte trafficking induced by SAH

**Fig. 3:**
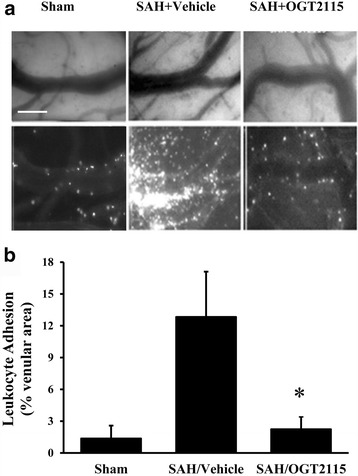
Inhibition of heparanase attenuates leukocyte adhesion in pial venules induced by subarachnoid hemorrhage (SAH). **a** Representative images showed the effect of heparanase inhibition (via OGT2115) on SAH-associated pial venular leukocyte trafficking at 48 h post-SAH. The contour of the pial venules was observed under the bright field (upper panels). The leukocyte adhesion was expressed as the bright area occupied by Rhodamine-6G-labeled leukocytes (lower panels). Scale = 50 μm. **b** Quantitative results of the effects of heparanase inhibitor (OGT2115) on SAH associated pial venular leukocyte adhesion in SAH. Leukocyte adhesion is expressed as the percentage of the viewed venular area occupied by rhodamined-6G-labeled leukocytes. In **b**, values are mean ± SEM, *n* = 4–6 per group

To investigate the inflammatory response to SAH, PVLA was observed through an intravital microscope. PVLA was expressed as the percentage of the pial venular area occupied by adherent R6G-labeled leukocytes at 48 h post-SAH. The representative images and quantitative results were shown in Fig. 3a, b, respectively. A markedly increase in PVLA was observed in vehicle-treated rats subjected to SAH at 48 h when compared to the sham surgical group (12.8 ± 4.3% *vs* 1.4 ± 1.2%). Treatment with heparanase inhibitor OGT2115 significantly reduced PVLA levels relative to vehicle (2.2 ± 1.2%).

### Heparanase inhibition improves neurologic scores after SAH

**Fig. 4:**
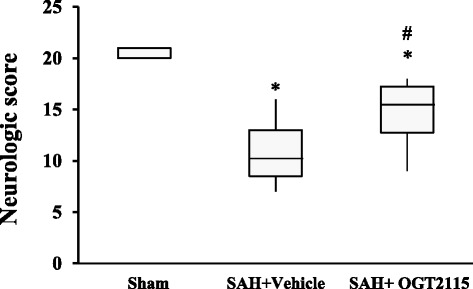
Inhibition of heparanase improves neurological score after subarachnoid hemorrhage (SAH). The data, presented in boxplot form, represents neurological scores obtained at 48 h of post-SAH, where the *upper* and *lower box* edges represent the 25th and 75th percentiles, and the whiskers represent the 95% confidence intervals. The median values are depicted by the *horizontal lines within the boxes*. Significant decrease in neurological score was observed in SAH rats. In contrast, application of heparanase inhibitor OGT2115 is associated with significantly increased neurological score. Values are mean ± SEM, *p < 0.05 *vs* sham, #*p* < 0.05 vs SAH+ vehicle control, *n* = 6 per group

The neurologic outcome scores for the sham, SAH, SAH + vehicle, or SAH + OGT2115 group are summarized in Fig. 4. Neurologic assessments were performed using the scoring system described in the “Methods” section. The maximum achievable score was 21, which translates to no functional deficit. The nonparametric data are presented as a boxplot, with median values for each group. Since both SAH group and SAH + vehicle group showed virtually no significant difference, the data of these two groups were pooled. Neurologic deficit scores of the sham surgical group were tightly distributed, ranging from 20 to 21. All the SAH-exposed rats displayed median neurologic scores that were significantly lower than in the sham group. However, a significant improvement of neurologic function was noted when the SAH + OGT2115 control group was compared with the SAH + vehicle group (11 ± 3 vs 15 ± 3; *p* <0.05) (Fig. 4).

## Discussion

In the present study, SAH in rat is produced via an endovascular perforation method that has been employed by many laboratories [39, 40]. This method has been characterized as one of the best models for investigating subarachnoid hemorrhage, since it mimics the rupture of human cerebral aneurysm and closely matches its clinical manifestation in terms of morbidity, mortality, and severity [41].

We observed that heparanase expression was increased during early brain injury following SAH and that heparanase was detected exclusively in neurons at 24 h post SAH. Moreover, inhibiting heparanase activity abolished SAH-induced leukocyte adhesion in pial venules. Heparanase upregulation has been considered as one of the mechanisms underlying the induction of angiogenesis during repeated hypoxic exposures and tumor metastasis [19, 42–46]. Navarro and colleagues showed that upregulation of both heparanase and vascular endothelial growth factor (VEGF) under repeated hypoxic conditions promoted angiogenesis [19]. Angiogenesis under pathological condition may represent a compensatory mechanism to promote blood flow to the ischemic, or injured, area of the brain. In particular, increased angiogenesis in the penumbra area attenuates ischemic neuronal cell death [44]. The higher density of blood vessels seems to correlate with better functional recovery, longer survival, and increased cerebral blood flow to areas surrounding the brain infarcts in stroke patients [47–49]. However, the role of heparanase in SAH-associated neuropathy is still unknown. Our current study is the first report to investigate the association between the upregulation of heparanase, cerebrovascular inflammation, and neurological impairment in SAH.

In the current study, we found that SAH induced significant leukocyte migration through the pial venule, indicating an inflammatory response following SAH. Interestingly, inhibition of heparanase (by OGT2115) significantly reduced leukocyte trafficking at 48 h post-SAH, suggesting that heparanase activation is involved in SAH-associated inflammatory reaction. It has been well known that heparanase cleaves heparan sulfate on HSPG, a major constituent of ECM that functions as a storage depot of numerous enzymes and cytokines [50], leading to ECM degradation, tissue invasion, and angiogenesis. As such, heparanase-induced impairment of the structural integrity of the ECM may lead to inflammation as represented by the promotion of leukocyte migration across the endothelium. This has been linked to a compensatory angiogenesis, which is considered beneficial in reperfusion post stroke. However, it may inadvertently lead to an inflammatory response and tissue injury in the brain. Sasaki and colleagues reported that heparanase expression on the cell surface of macrophages was essential for its leukocyte extravasation through the basement membrane [21]. In a recent study, Level et al. also found that heparanase augmented the adhesion of neutrophils and mononuclear cells to vascular endothelial cells in a concentration-dependent manner [51]. Heparanase exists as a 65-kDa inactive precursor form and a 50-kDa active form. Both forms can mediate cell adhesion via a β1-integrin-dependent mechanisms that is unrelated to their enzymatic activity [52]. However, enzymatic activity of heparanase, or at least the ability to be processed to an enzymatically active form, is still essential for its proinflammatory activity in vivo [51]. Consistent with this finding, our preliminary experimental results showed an elevation of active form of heparanase expression in the ipsilateral cortex at 24 h after SAH (data not shown), which is suggestive of the contributory role of heparanase in SAH-induced cerebrovascular inflammation. This issue needs further investigation.

We further investigated the role of heparanase in SAH-related early neurological dysfunction. Based on our previous experience, the neurological function cannot be assessed accurately within 24 h post-surgery due to the stress and residual influence from either anesthesia and/or surgical procedure. Stable and more reliable neurological scores can only be obtained at 48 h, which was our earliest evaluation time point during this study. Nevertheless, we found that inhibition of heparanase by OGT2115 within the first 24 h post-SAH prevented SAH-associated neurological deficits at 48 h post-SAH, suggesting an important role of heparanase in mediating early brain injury following SAH.

Our previous findings indicate that blocking SAH-induced leukocyte adhesion and transmigration into the brain parenchyma improves neurological outcome [37]. In SAH, the initial insult induces an inflammatory state characterized by early recruitment of leukocytes and increased production of cytokines and chemokines [1, 53]. This immune response correlates with SAH severity and has been linked to neural cell injury, BBB dysfunction, and delayed cerebral ischemia [54–57]. Therefore, the enhanced leukocyte trafficking may be an important prerequisite in promoting neuropathology that are likely to be an effective treatment target to prevent SAH-associated neurological impairment.

There are limitations of the present study. First, in this study, early application of heparanase inhibitor resulted in significant suppression of SAH-associated cerebrovascular inflammation and improvement of neurological function. However, the long-term prognosis of this intervention was not addressed. Considering that heparanase promotes angiogenesis and that angiogenesis provides neuroprotection against ischemic brain damage, a delayed complication of SAH, it is of great importance to assess the efficacy of heparanase inhibition over the late phase of SAH. As such, an extended observation window is needed. Another limitation of this study is that the localization (i.e., the cellular and/or compartmental specificity) of heparanase expression needs to be emphasized. Navarro et al. reported [19] that, after ischemic stroke, the upregulation of heparanase expression was localized predominately in neurons and not in astrocytes, microglia, or endothelium. Another study, however, identified that heparanase expression was confined to the endothelial cells at 7 days, but then shifted to astrocytes at 14 days post ischemia [16]. Takahashi et al. also detected heparanase expression only in astrocytes after the subacute phase (3–7 days) post brain ischemia. Apparently, expression of heparanase correlates temporally and spatially in different cell types in the brain in different stroke models [16, 19, 32].Consistent with these findings, in our current study, a significant population of heparanase-positive cells in the SAH brain sections were not positive for NeuN staining, indicating SAH-associated heparanase expression in cells other than neurons (Fig. 2.). Nevertheless, our experiments focused on the early phase of SAH within 24 h; further investigation is warranted to investigate other time points both in the early and delayed phases of SAH to delineate the regulation of heparanase expression.

## Conclusions

Taken together, the present findings show a significant elevation of SAH-associated heparanase expression that is linked to post-SAH leukocyte trafficking. Selective blocking heparanase by OGT2115 attenuates cerebrovascular inflammation and prevents SAH-induced neurological impairment, suggesting an involvement of heparanase in SAH-associated neuropathy.

## Abbreviations

aCSF: Artificial Cerebrospinal fluid
ANOVA: Analysis of variance
CCA: Common carotid artery
DCI: Delayed cerebral ischemia
ECA: External carotid arteries
HS: Heparan sulfate
HSPG: HS proteoglycans
ICA: Internal carotid artery
ICP: Intracranial pressure
LDF: Laser doppler flowmetry
MAP: Mean arterial pressure
MCA: Middle cerebral artery
PVLA: Pial venule intravascular leukocyte adhesion
R6G: Rhodamine-6G
rCBF: Regional cerebral blood flow
SAH: Subarachnoid hemorrhage
